# Large-Scale Analysis of Apolipoprotein CIII Glycosylation by Ultrahigh Resolution Mass Spectrometry

**DOI:** 10.3389/fchem.2021.678883

**Published:** 2021-05-07

**Authors:** Daniel Demus, Annemieke Naber, Viktoria Dotz, Bas C. Jansen, Marco R. Bladergroen, Jan Nouta, Eric J. G. Sijbrands, Mandy Van Hoek, Simone Nicolardi, Manfred Wuhrer

**Affiliations:** ^1^ Leiden University Medical Center, Center for Proteomics and Metabolomics, Leiden, Netherlands; ^2^ Ludger Ltd., Culham Science Centre, Abingdon, United Kingdom; ^3^ Department of Internal Medicine, Erasmus University Medical Center, Rotterdam, Netherlands

**Keywords:** high-throughput, mass spectrometry, apolipoprotein-CIII, glycosylation, oxidation

## Abstract

Apolipoprotein-CIII (apo-CIII) is a glycoprotein involved in lipid metabolism and its levels are associated with cardiovascular disease risk. Apo-CIII sialylation is associated with improved plasma triglyceride levels and its glycosylation may have an effect on the clearance of triglyceride-rich lipoproteins by directing these particles to different metabolic pathways. Large-scale sample cohort studies are required to fully elucidate the role of apo-CIII glycosylation in lipid metabolism and associated cardiovascular disease. In this study, we revisited a high-throughput workflow for the analysis of intact apo-CIII by ultrahigh-resolution MALDI FT-ICR MS. The workflow includes a chemical oxidation step to reduce methionine oxidation heterogeneity and spectrum complexity. Sinapinic acid matrix was used to minimize the loss of sialic acids upon MALDI. MassyTools software was used to standardize and automate MS data processing and quality control. This method was applied on 771 plasma samples from individuals without diabetes allowing for an evaluation of the expression levels of apo-CIII glycoforms against a panel of lipid biomarkers demonstrating the validity of the method. Our study supports the hypothesis that triglyceride clearance may be regulated, or at least strongly influenced by apo-CIII sialylation. Interestingly, the association of apo-CIII glycoforms with triglyceride levels was found to be largely independent of body mass index. Due to its precision and throughput, the new workflow will allow studying the role of apo-CIII in the regulation of lipid metabolism in various disease settings.

## Introduction

Lipid metabolism is regulated by complex biological mechanisms in which apolipoproteins – proteins embedded in lipoprotein particles – modulate the transport and availability of blood lipids (Mahley et al., 1984). Apolipoprotein-CIII (apo-CIII) is a 79 amino acid glycoprotein present on the surface of triglyceride-rich lipoproteins and is an inhibitor of lipoprotein lipase (LPL), an enzyme that hydrolyzes triglycerides into fatty acids (Shachter, 2001; Larsson et al., 2013, 2017). Apo-CIII has been associated with increased monocyte adhesion to the endothelium (Kawakami et al., 2006) and enhanced binding of apoB-containing lipoproteins to vascular proteoglycans (Olin-Lewis et al., 2002). High apo-CIII levels are associated with hypertriglyceridemia (Kohan, 2015; Dai et al., 2019; Taskinen et al., 2019) and increased cardiovascular disease risk in the general population (Wyler Von Ballmoos et al., 2015; Rosenson et al., 2016; Dai et al., 2019) and diabetes mellitus (Juntti-Berggren and Berggren, 2017; Christopoulou et al., 2019). Recently, the clinical interest for this protein has increased due to the promising results obtained from antisense oligonucleotide-based therapies for the reduction of apo-CIII and triglyceride levels (Pollin et al., 2008; Rocha et al., 2017; Reyes-Soffer et al., 2019; Taskinen et al., 2019).

Apo-CIII exists in four major proteoforms: one non-glycosylated form (apo-CIII_0a_) and three O-glycosylated variants with a core 1 (T-antigen) glycan structure, which is either non-sialylated (apo-CIII_0c_), monosialylated (apo-CIII_1_) or disialylated (apo-CIII_2_) (Nedelkov, 2017; Ramms and Gordts, 2018). Low-abundance fucosylated, non-sialylated apo-CIII forms have also been described (Nicolardi et al., 2013a). It has been shown that not only the levels of apo-CIII but also the specific glycoforms and their relative expression control triglyceride metabolism (Yassine et al., 2015; Koska et al., 2016). For example, an inverse association between apo-CIII_2_/apo-CIII_1_ ratio and triglyceride levels has been confirmed by two independent studies (Koska et al., 2016; Kegulian et al., 2019). It has also been shown that sialylation modulates the apo-CIII affinity for hepatic receptors that clear lipoprotein particles (Kegulian et al., 2019) and that different proteoforms of apo-CIII may affect the inhibition of LPL (Holleboom et al., 2011) and the interaction of LDL with the vascular wall (Hiukka et al., 2009). Since the association of different apo-CIII proteoforms with specific cardiometabolic endpoints has not been fully elucidated, further research in large sample cohorts is warranted.

We have developed a high-throughput method based on magnetic-bead extraction and matrix-assisted laser desorption/ionization (MALDI) and ultrahigh-resolution Fourier transform ion cyclotron resonance (FT-ICR) mass spectrometry (MS) for the analysis of serum apo-CIII proteoforms (Nicolardi et al., 2013a; Nicolardi et al., 2013b). Apo-CIII contains methionine residues, which can be (partially) oxidized during biological processes *in vivo* (Stadtman et al., 2003), sample processing and freeze-thaw cycles (Borges et al., 2014). The presence of different oxidoforms increases mass spectra complexity, which complicates MS data processing and affects the repeatability of measurements. Although the analyte oxidation may not pose a serious challenge in MALDI MS analysis of single samples, it can seriously impact the precision and accuracy of quantitative measurements in large sample cohorts.

In the current study, we have applied a modified workflow employing a previously established MALDI FT-ICR MS method preceded by a chemical oxidation step for complete oxidation of apo-CIII methionine residues. This results in highly reproducible high-throughput measurements for relative quantification of apo-CIII proteoforms in a large number of plasma samples varying in protein oxidation levels. Furthermore, we have adopted sinapinic acid (SPA) as a MALDI matrix to minimize the loss of sialic acid induced by MALDI. The high-throughput quantitation software, MassyTools (Jansen et al., 2015), was here further developed to facilitate semi-automated MS data processing for intact proteins. The validity of the new workflow was tested on a clinical cohort comprised of 771 plasma samples, which allowed the evaluation of the relationship between apo-CIII glycoforms and metabolic biomarkers, such as BMI, cholesterol, and triglyceride levels.

## Materials and Methods

### Clinical Samples

Blood plasma samples from a group of individuals without diabetes of the DiaGene Study were used. The DiaGene Study is a case-control study comprising 1886 type-2 diabetes patients and 854 controls without diabetes, from the areas of Eindhoven and Veldhoven, in the Netherlands. The study is described in detail elsewhere (Van Herpt et al., 2017). For the current study, after quality control, apo-CIII glycosylation data were available for 771 samples, in 746 whereof, data on clinical characteristics were available. All participants gave their written informed consent. This study was approved by the Medical Ethics Committees of the Erasmus University Medical Center, Catharina Hospital and Maxima Medical Center.

Clinical information and blood samples were obtained at baseline, as described previously (Van Herpt et al., 2017). Triglycerides and cholesterol concentrations were measured using standard clinical chemistry essays and reported by the collecting clinic. Non-high-density lipoprotein (non-HDL)-cholesterol was calculated by subtracting the high-density lipoprotein (HDL)-cholesterol from the total cholesterol, body mass index (BMI) was calculated by dividing the body mass (in kg) by the square of the body length (in m). Triglyceride concentrations were logarithmically transformed before linear regression analysis, because of non-normal distribution.

### Chemicals

Magnetic beads (Dynabeads RPC-18) were purchased from Invitrogen Dynal AS, Oslo, Norway. VisuCon-F plasma standard from Affinity Biologicals, Ancaster, Canada. Hydrogen peroxide 30%, ethanol and acetone were purchased from Merck, Darmstadt, Germany. Acetonitrile (ACN) was from Biosolve Chimie SARL, France. Trifluoroacetic acid (TFA) was purchased from Thermo Fisher Scientific, Tewksbury, MA. Sinapinic acid (SPA) and α-Cyano-4-hydroxycinnamic acid (HCCA) from Sigma-Aldrich. Ultrapure milliQ water (18 MΩ cm at 25°C) was used throughout.

### High-Throughput RP-C18 Solid-Phase Extraction of Plasma Proteins

Plasma standards (VisuCon-F) were randomized over cohort sample plates. 10 µL of human blood plasma was transferred from the cohort sample plates into 96-well skirted PCR plates (4ti-0960/C, 4titude, Dorking, United Kingdom). 15 µL of an oxidizing solution (12% H_2_O_2_/0.5% TFA in water) was added to each sample. The plate was sealed with a pierce foil seal (4ti-0521, 4titude Ltd., Wotton, Surrey, United Kingdom) and incubated for 1 h at 37°C. Subsequently, the plate was cooled at 4°C for 30 min and centrifuged briefly at 800 × *g*. The pierce foil was removed and the plate was transferred onto a liquid handling robot (Hamilton, Bonaduz, Switzerland) where solid-phase extraction (SPE) was carried out as follows: the RP-C18 beads were activated by three washes using acetonitrile (ACN) and trifluoroacetic acid (TFA) solution in water (first wash using 50% ACN/0.1% TFA followed by two washes with 0.1% TFA). Next, plasma samples were transferred to the activated beads and incubated for 10 min at room temperature. The incubation was followed by three washes: one wash using 15% ACN and two washes with 0.1% TFA. Proteins were eluted by adding 15 µL of 50% ACN/0.1% TFA in water and incubating for 5 min at room temperature. For MALDI spotting, 2 μL of sample eluates were mixed with either 16 μL of sinapinic acid solution (1.3 g/L in 2:1 v/v ethanol/acetone) or 15 μL alpha-cyano-4-hydroxycinnamic acid solution (1.4 g/L in 2:1 v/v ethanol/acetone). 1.5 μL of each sample mix was spotted in duplicate onto a MALDI AnchorChip target plate (800 μm anchor diameter; Bruker Daltonics, Bremen, Germany) and allowed to air-dry before MALDI MS analysis.

### MALDI FT-ICR Mass Spectrometry and MS Data Analysis

All MALDI MS experiments were performed on a 15 T solariX XR FT-ICR mass spectrometer (Bruker Daltonics) equipped with a Smartbeam II™ laser system (355 nm wavelength) and a ParaCell detector. All spectra were acquired in the *m/z*-range 3495–30,000, from the average of ten scans of 200 laser shots (at 500 Hz) each using 524,288 data points. The analyzer parameters were set as previously reported (Van Der Burgt et al., 2019). Briefly, measurements were performed with high trapping potentials (up to 8.5 V) and high ParaCell DC biases (up to 8.8 V) and with a Sweep excitation power of 57% for 13.5 µs. A laser power of 20% and “medium” laser focus was used for MS measurements using HCCA, while a laser power of 30% and “ultra-large” focus was used with SPA. Details on MS data processing and statistical analysis can be found in Supplementary Material (Additional experimental details: MS data processing and statistical analysis).

## Results and Discussion

### The Controlled Oxidation of Methionine Residues Reduces the Complexity of Mass Spectra

A common event observed in proteomics studies is the oxidation of methionine residues due to biological and pathological processes occurring *in vivo* (Stadtman et al., 2003), sample storage and processing (Borges et al., 2014). These reactions are so common that, in bottom-up studies, methionine oxidation is often included in the database search as a variable or even fixed modification. However, in general, the peptides generated by enzymatic digestion (e.g. using trypsin) are small and often do not contain methionine residues and although a (partial) oxidation of methionine residues increases the number of peptides in a digest, these unwanted reactions do not significantly affect the analysis (Lao et al., 2015; Hains and Robinson, 2017; Bettinger et al., 2020). In a clinical setting, the use of fresh samples may be an ideal approach, especially for diagnostic purposes based on profiling of intact protein. Whereas in cohort studies, the collection of large numbers of clinical samples, their storage, transfer between institutions and multiple use may lead to oxidation processes that affect the analysis of intact proteins by increasing the heterogeneity of proteoforms detected in a spectrum. The higher complexity increases the chance of overlapping signals and reduces the sensitivity of the measurements due to the spreading of the signal over a higher number of species. This results in MS spectra characterized by the presence of interfering species and very low abundant analyte peaks, which do not meet acceptable spectral quality criteria for consideration in the statistically significant quantitative analysis.

Apo-CIII contains two methionine residues which can be oxidized to form methionine sulfoxide (MetO) and methionine sulfone (MetO_2_) although this latter form requires harsher oxidizing conditions (Kim et al., 2015; Lim et al., 2019). Previously, MALDI-TOF MS methods have been used in analyses of apo-CIII proteoforms (Trenchevska et al., 2016). In such low-resolution methods, apo-CIII oxidoforms cannot be resolved, however, their presence results in the broadening and distortion of apo-CIII proteoforms’ signals, which can eventually overlap or interfere with signals of other proteins affecting their quantification. The chance of signal interference increases when SPE is used for the enrichment of apo-CIII as it leads to the co-enrichment of other small plasma proteins. The application of more specific enrichment methods, such as immunocapture, may help to reduce signals interfering with the various apo-CIII oxidoforms, but was not implemented in the present study for simplicity reasons. Of note, apo-CIII proteoforms have been analyzed by methods employing LC systems (Kailemia et al., 2018; Olivieri et al., 2018). Despite certain advantages over MALDI-TOF MS, such as absolute quantification and enhanced resolution, the throughput of this approach remains relatively low.

**FIGURE 1:**
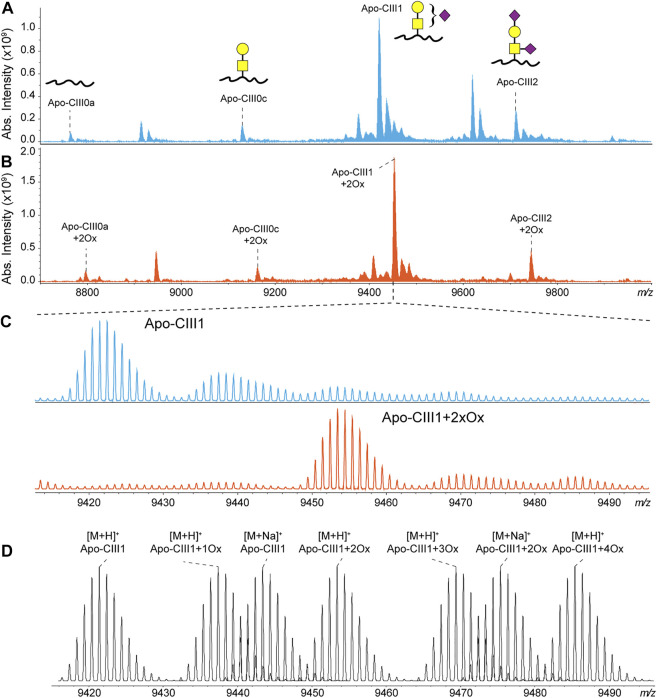
MALDI FT-ICR mass spectra of apo-CIII proteoforms from **(A)** non-treated (blue) and **(B)** H_2_O_2_ treated (orange) human blood plasma samples. The four most abundant proteoforms of apo-CIII are annotated. Mass spectra from non-treated samples are characterized by the presence of oxidoforms as exemplified for apo-CIII1 in panel **(C)**. The H_2_O_2_ treatment allows the almost complete conversion of methionine residues to methionine sulfoxide with a minor conversion to methionine sulfone. Theoretical isotopic distribution of apo-CIII1 proteoforms are depicted in panel **(D)**. For graphical representations of glycan structures: yellow square (N-acetylgalactosamine), yellow circle (galactose), purple diamond (N-acetylneuraminic acid).

Recently, we have developed a method for the analysis of apo-CIII proteoforms using ultrahigh-resolution MALDI FT-ICR MS (Nicolardi et al., 2013a). Apo-CIII proteoforms were mainly detected as singly charged ions. Thus, apo-CIII oxidoforms (1 and 2 times MetO) were detected at +15.995 Th and +31.990 Th from the non-oxidized forms (Figure 1). The degree of methionine oxidation of apo-CIII can vary greatly but the complete oxidation of apo-CIII (i.e. 100% conversion of the two methionine residues to MetO) is not commonly observed (Supplementary Figure S1). Therefore, for each of the four major proteoforms of apo-CIII, two additional oxidoforms were observed in MALDI FT-ICR MS spectra resulting in twelve proteoforms (Figure 1). In addition to that, we were able to detect C-terminal alanine cleaved and fucosylated proteoforms (Supplementary Table S1).

To reduce sample complexity, we included an oxidation step with hydrogen peroxide to perform a controlled oxidation of both apo-CIII methionine residues to MetO (Figure 1). While the implementation of the oxidation step added 2 h to the workflow for the analysis, it reduced the heterogeneity of the spectra and facilitated MS data processing using MassyTools software (see Implementation of MassyTools software for high-throughput MS data processing) (Jansen et al., 2015). The efficiency of the controlled oxidation was tested on 136 standard and 771 clinical plasma samples. The relative intensities between the non-, mono- and di-oxidized forms of apo-CIII_0a_, apo-CIII_0c_, apo-CIII_1_, and apo-CIII_2_ are reported in Supplementary Tables S2, S3. Oxidation rates over 90% were found for apo-CIII_1_ and apo-CIII_2_. Oxidation efficiency seemed to be lower for apo-CIII_0a_, apo-CIII_0c,_ however, close inspection of the spectra revealed the presence of interfering species that contributed to the signal of the non- and mono-oxidized forms of apo-CIII_0a_, apo-CIII_0c_ thus increasing their apparent relative intensity (Supplementary Figure S2). Therefore, the controlled oxidation was considered efficient for all four proteoforms by providing consistent oxidation rates across standard and clinical plasma samples. These results supported our strategy of using only the signal of the di-oxidized apo-CIII proteoforms for further statistical analysis. The good efficiency and repeatability of the oxidation step allowed us to assess associations between apo-CIII glycosylation and different lipid markers using only the signal of the di-oxidized forms.

### Minimizing Sialic Acid Loss Using Sinapinic Acid as MALDI Matrix

**FIGURE 2:**
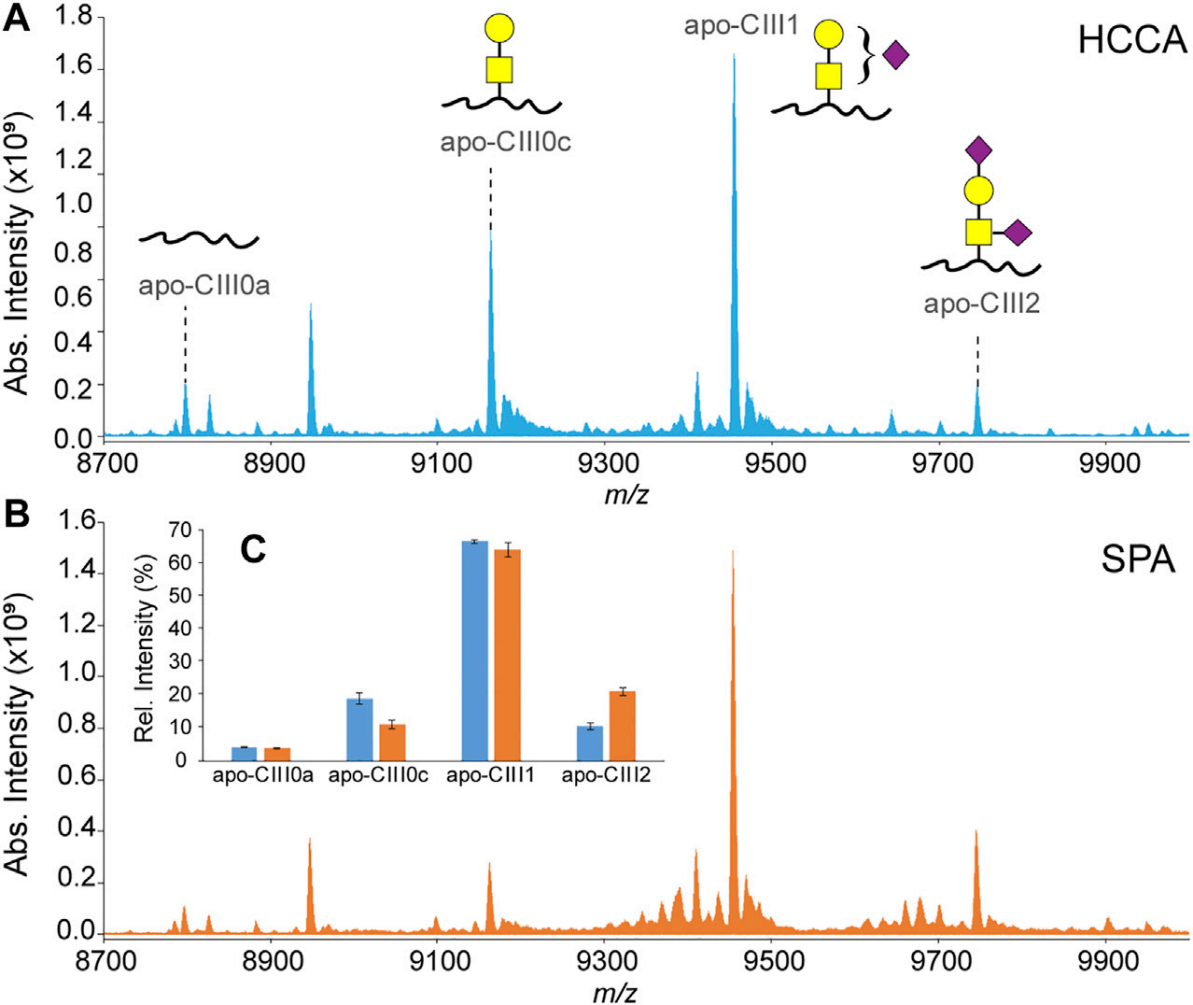
MALDI-FT-ICR mass spectra of apo-CIII proteoforms from H_2_O_2_-treated human blood plasma samples obtained using either HCCA **(A)** or SPA **(B)** as MALDI matrices. Relative peak intensities of the four most abundant proteoforms of apo-CIII are shown for both sets (n = 3 in duplicate) of samples in the inset **(C)**.

In our previously reported ultrahigh-resolution MALDI FT-ICR MS method for the analysis of apo-CIII proteoforms HCCA was used as a MALDI matrix (Nicolardi et al., 2013a; Nicolardi et al., 2013b). This compound was chosen to increase the sensitivity for other serum peptides and small proteins present in C18-SPE eluates obtained from the high-throughput enrichment step using magnetic beads. However, it is known that sialic acid loss can result from in-source decay fragmentation events of glycan structures even when linked to peptides and proteins. In fact, previous reports on the analysis of apo-CIII by MALDI-TOF MS were based on the use of a MALDI matrix colder than HCCA, namely SPA (Kegulian et al., 2019; Koska et al., 2016; Yassine et al., 2015). The use of SPA allowed to minimize the loss of sialic acid, as evidenced by an increased relative intensity of both the mono- and the disialylated apo-CIII proteoforms and leading to reproducible apo-CIII glycosylation profiles (Figure 2; Supplementary Table S4). Importantly, compared to other matrices previously used for profiling of apo-CIII glycoforms such as DHB (Palmigiano et al., 2017; Wada and Okamoto, 2020), SPA provides more desirable matrix/analyte co-crystallization in the context of high-throughput, automated MALDI measurements.

### Implementation of MassyTools Software for High-Throughput MS Data Processing

One of the advantages of using ultrahigh-resolution MS is that measurements at isotopic resolution provide more spectra information compared to broad-peak detection in linear mode MALDI-TOF MS. Previously, we showed that the goodness of the observed isotopic distributions can be used as a quality control parameter for the selection of high-quality spectra generated from the analysis of a large cohort of samples (Nicolardi et al., 2010). This concept was then implemented in a more powerful software–namely, MassyTools–developed for the high-throughput processing of MALDI mass spectra (Jansen et al., 2015). MassyTools allows the determination of a series of quality control parameters that can be used to perform a curation of MS data at different levels. Mass spectra with unacceptable internal calibration quality and low intensity were discarded at first. Then, the quality of the signal of each apo-CIII proteoform was assessed using the S/N and MME values determined for the most intense peak within an isotopic distribution. Additionally, the quality of such distribution (i.e. IPQ value) was taken into account. The distributions of values of these parameters over 136 standard and 771 clinical plasma samples are reported in Supplementary Figures S3, S4; Supplementary Table S5. The analytes passing the curation process were then used for statistical analysis.

As assessed on 136 standard plasma samples, which were distributed over 17 MALDI target plates measured over 28 days, the method provided good repeatability for relative quantitation of all four proteoforms with CVs in a range of 1–18% for average intra-plate and 6–16% for inter-plate variability (Table 1). While we reduced in-source decay by selecting SPA as a MALDI matrix, we expect that partial sialic acid loss from apo-CIII_1_ during MS analysis may lead to a slight, artificial increase in the apo-CIII_0c_ glycoform abundance. Hence, fluctuations in the extent of sialic acid loss may contribute to the larger CVs for apo-CIII_0c_.

**TABLE 1:** Inter- and intra-plate variability. Relative peak intensities, standard deviation (SD) and coefficient of variation (CV) are given for the intra- and inter-plate variability based on 136 plasma standards.

		Apo-CIII_0a_ +2Ox	Apo-CIII_0c_ +2Ox	Apo-CIII_1_ +2Ox	Apo-CIII_2_ +2Ox
Inter-plate	Relative peak intensity	0.039	0.114	0.636	0.203
	SD	0.003	0.018	0.04	0.02
	CV	8%	16%	6%	10%
Average intra-plate	CV	7%	18%	1%	10%

### Associations Between Apo-CIII Sialylation and Lipid Markers

We used this approach to determine non-glycosylated and the glycosylated non-sialylated, mono-sialylated and disialylated apo-CIII glycoforms within a cohort of 746 individuals without diabetes (cohort characteristics in Table 2) and test their association with a range of metabolic biomarkers. We found the association of disialylated apo-CIII_2_ with overall improved lipid profiles and decreased BMI (Table 3; Supplementary Table S6), which is in accordance with some of the previous reports (Koska et al., 2016; Kegulian et al., 2019). A subgroup analysis in participants not using statins or fibrates, did not change these associations (Supplementary Tables S7A,B).

**TABLE 2:** DiaGene cohort characteristics.

	Individuals without diabetes
Participants, n	746
Male sex, n (%)	290 (38.9)
Age, year	65.7 (6.7)
Age within males, year	66.0 (6.7)
Age within females, year	65.5 (6.7)
BMI, kg/m^2^	25.4 (4.5)
HDL-cholesterol, mmol/l	1.48 (0.36)
non-HDL-cholesterol, mmol/l	4.09 (0.97)
LDL-cholesterol, mmol/l	3.56 (0.90)
Triglycerides, mmol/l	1.20 (0.68)
Total cholesterol, mmol/l	5.57 (0.99)
Use of lipid lowering therapy, n (%)	93 (12.5)

Mean (and standard deviation) for normal distribution and median (and interquartile range) for non-normal distributions (BMI and triglycerides).

**TABLE 3:** Associations of apo-CIII glycosylation with clinical characteristics.

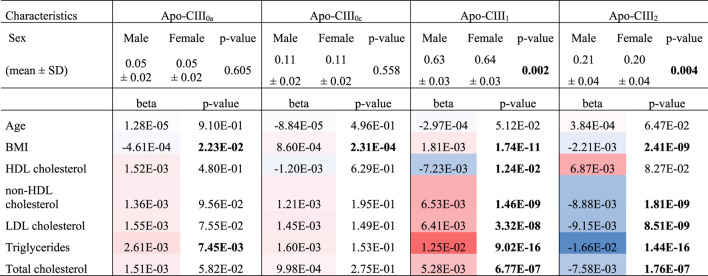

Blue: negative associations, red: positive associations, bold: significant *p*-value. *p*-values of logarithmically transformed triglyceride concentrations, beta of non-transformed concen-trations, bold values ‐ significant p-values.

**FIGURE 3:**
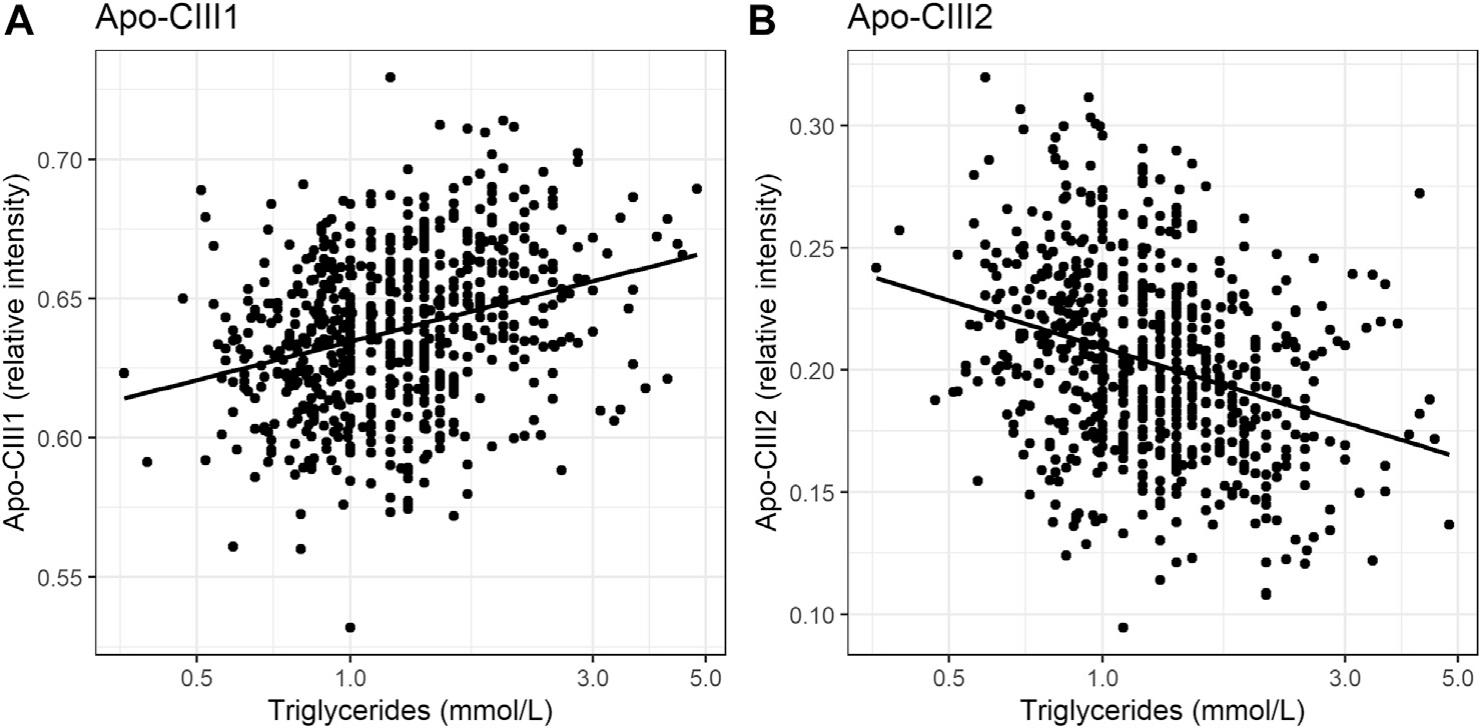
Relationship of apo-CIII1 [panel **(A)**] and apo-CIII2 [panel **(B)**] with triglycerides.

So far, the inhibitory effect of apo-CIII on LPL has been linked to total apo-CIII concentration, but not to the relative proportion of apo-CIII glycoforms (Olivieri et al., 2018). Recent studies proposed that the presence of apo-CIII on triglyceride-rich lipoproteins (TRLs) alters the affinity between TRLs and their receptors in the liver. Kegulian *et al.* demonstrated that the degree of apo-CIII sialylation directs TRLs to different hepatic clearance pathways, as shown in mice (Kegulian et al., 2019). In detail, apo-CIII_1_-enriched very low-density lipoproteins (VLDLs) are preferentially cleared by faster-acting low-density lipoprotein (LDL) receptor (LDLR) and LDL receptor-related protein 1 (LRP1), whereas apo-CIII_2_ directs VLDLs to syndecan 1 (SDC1) receptors that are characterized by a slower but larger capacity metabolism of TRLs. The same study also showed that a 13 weeks antisense oligonucleotide treatment for apo-CIII, which, as expected, reduced plasma TG levels, also altered relative abundances of these two glycoforms leading to an increase of apo-CIII_2_ and a decrease of apo-CIII_1_. The increase of the apo-CIII_2_/apo-CIII_1_ ratio in a response to the antisense oligonucleotide therapy was explained by a differing capacity and clearance speed of the hepatic TRL receptors. In support of this, we observed in our cohort study of individuals without diabetes a positive association of the relative abundance of apo-CIII_1_ glycoform with TG levels, and a negative association for apo-CIII_2_ (Figure 3; Table 3).

Our study supports the hypothesis that triglyceride clearance may be regulated, or at least strongly influenced, by apo-CIII glycosylation, specifically sialylation. However, other aspects have to be considered. For instance, defective LDLR/LRP1-driven metabolic pathways might lead to decreased clearance of TGs. Expression and stability of LDLR and LRP1 in the liver might be affected by naturally occurring genetic variants (Oldoni et al., 2018; Paththinige et al., 2018; Reyes-Soffer et al., 2019; Xu et al., 2020). Moreover, it has been shown in mice that a high-fat diet can lead to the down-regulated expression of hepatic LRP1 by causing hyperglycemia with a high level of plasma triglycerides (Kim et al., 2014). In humans, obesity is associated with increases in plasma triglycerides (Howard et al., 2003; Franssen et al., 2011). We hypothesized that the association of apo-CIII glycoforms with triglycerides could be confounded by BMI. Surprisingly, after adjustment for BMI, the direction of effect and goodness-of-fit did not evidently change (Supplementary Tables S8A,B). This indicates that the association of apo-CIII sialylation with triglycerides is largely independent of BMI, and that it is not obesity-associated physiological changes that determine apo-CIII sialylation and its association with triglycerides.

Expression levels of apo-CIII were not investigated in this study. The differences in apo-CIII glycosylation profiles observed between individuals may be caused by varying expression levels of apo-CIII (Olivieri et al., 2018) or apo-CIII glycoforms (Holleboom et al., 2011), or the accumulation of certain glycoforms due to dysfunctional clearance pathways, based on recent findings by Kegulian et al. (Kegulian et al., 2019). It may also be a combination of the listed factors, which should be explored in further research. Nevertheless, from the results of this study, we cannot determine whether apo-CIII sialylation influences triglyceride levels, or vice versa. Further studies are needed to elucidate the genetic and environmental factors that determine apo-CIII sialylation in health and disease.

## Conclusion

Apo-CIII is a novel potential drug target in the management of cardiovascular disease driven by multiple studies demonstrating that plasma levels of apo-CIII are predictive of coronary heart disease and the risk of disease-related events (Borén et al., 2020). Previously, it has been shown that sialylated apo-CIII glycoforms are differentially cleared by hepatic receptors and that a higher apo-CIII_2_/apo-CIII_1_ ratio is associated with improved triglyceride levels (Kegulian et al., 2019). In humans, the production rates of these two glycoforms are comparable (Mauger et al., 2006), therefore varying apo-CIII2/apo-CIII1 ratios between individuals in healthy and disease groups might suggest various dysfunctional mechanisms involved in their production and clearance. This is the first large-scale study of apo-CIII glycosylation by ultrahigh resolution mass spectrometry. Clinical cohort studies employing large numbers of individuals will provide more insight into this topic, and the development of highly robust and accurate analytical methods enabling such large-scale studies is warranted.

Here, we present a workflow for high-throughput MALDI FT-ICR MS analysis of apo-CIII glycosylation in human plasma samples varying in protein oxidation levels. The controlled oxidation of apo-CIII methionine residues, the use of sinapinic acid as a MALDI matrix, and the use of MassyTools software for semi-automated, standardized spectra processing have been implemented to achieve highly repeatable measurements of intact apo-CIII proteoforms. The new analytical workflow allowed us to overcome the problem of the high spectral heterogeneity produced by methionine oxidation thus allowing the robust screening of a large cohort of plasma samples for the relative quantitation of apo-CIII proteoforms. Importantly, the evaluation of MS spectra-derived quality parameters was implemented to minimize biases and ensure accuracy of collected data.

The cohort analysis confirmed that the level of apo-CIII sialylation is strongly associated with lipid biomarkers, especially with triglyceride levels. The relation between relative abundances of apo-CIII glycoforms and cardiovascular disease development should be further explored. More insight into the role of apo-CIII glycosylation in disease pathophysiology could provide new drug targets. Also, understanding of the mechanisms of existing drugs might increase by considering apo-CIII glycosylation. The methods presented, will enable such large-scale studies.

## Data Availability

The datasets generated for this study are available on request to the corresponding author.

## References

[B1] Bettinger J. Q. Welle K. A. Hryhorenko J. R. Ghaemmaghami S. (2020). Quantitative Analysis of In Vivo Methionine Oxidation of the Human Proteome. J. Proteome Res. 19 (2), 624–633. 10.1021/acs.jproteome.9b00505 31801345 PMC7077757

[B2] Borén J. Packard C. J. Taskinen M. R. (2020). The Roles of ApoC-III on the Metabolism of Triglyceride-Rich Lipoproteins in Humans. Front. Endocrinol. 11 (July), 1–10. 10.3389/fendo.2020.00474 PMC7399058 32849270

[B3] Borges C. R. Rehder D. S. Jensen S. Schaab M. R. Sherma N. D. Yassine H. (2014). Elevated Plasma Albumin and Apolipoprotein A-I Oxidation under Suboptimal Specimen Storage Conditions. Mol. Cell Proteomics 13 (7), 1890–1899. 10.1074/mcp.m114.038455 24736286 PMC4083123

[B4] Christopoulou E. Tsimihodimos V. Filippatos T. Elisaf M. (2019). Apolipoprotein CIII and Diabetes. Is There a Link? Diabetes/Metabolism Res. Rev. 35 (3), e3118. 10.1002/dmrr.3118 30557902

[B5] Dai W. Zhang Z. Yao C. Zhao S. (2019). Emerging Evidences for the Opposite Role of Apolipoprotein C3 and Apolipoprotein A5 in Lipid Metabolism and Coronary Artery Disease. Lipids Health Dis. 18 (1), 1–7. 10.1186/s12944-019-1166-5 31836003 PMC6909560

[B6] Franssen R. Monajemi H. Stroes E. S. G. Kastelein J. J. P. (2011). Obesity and Dyslipidemia. Med. Clin. North America 95 (5), 893–902. 10.1016/j.mcna.2011.06.003 21855698

[B7] Fritz B. V. Ruotolo G. Robbins D. C. (2003). Obesity and Dyslipidemia. Endocrinol. Metab. Clin. of North Am 32 (4), 855–867. 10.1016/s0889-8529(03)00073-2 14711065

[B8] Hains P. G. Robinson P. J. (2017). The Impact of Commonly Used Alkylating Agents on Artifactual Peptide Modification. J. Proteome Res. 16 (9), 3443–3447. 10.1021/acs.jproteome.7b00022 28799334

[B9] Hiukka A. Ståhlman M. Pettersson C. Levin M. Adiels M. Teneberg S. (2009). ApoCIII-enriched LDL in Type 2 Diabetes Displays Altered Lipid Composition, Increased Susceptibility for Sphingomyelinase, and Increased Binding to Biglycan. Diabetes 58 (9), 2018–2026. 10.2337/db09-0206 19502413 PMC2731525

[B10] Holleboom A. G. Karlsson H. Lin R.-S. Beres T. M. Sierts J. A. Herman D. S. (2011). Heterozygosity for a Loss-Of-Function Mutation in GALNT2 Improves Plasma Triglyceride Clearance in Man. Cel Metab. 14 (6), 811–818. 10.1016/j.cmet.2011.11.005 PMC3523677 22152306

[B11] Jansen B. C. Reiding K. R. Bondt A. Hipgrave Ederveen A. L. Palmblad M. Falck D. (2015). MassyTools: A High-Throughput Targeted Data Processing Tool for Relative Quantitation and Quality Control Developed for Glycomic and Glycoproteomic MALDI-MS. J. Proteome Res. 14 (12), 5088–5098. 10.1021/acs.jproteome.5b00658 26565759

[B12] Juntti-Berggren L. Berggren P. O. (2017). Apolipoprotein CIII Is a New Player in Diabetes. Curr. Opin. Lipidol. 28 (1), 27–31. 10.1097/MOL.0000000000000372 27875339

[B13] Kailemia M. J. Wei W. Nguyen K. Beals E. Sawrey-Kubicek L. Rhodes C. (2018). Targeted Measurements of O- and N-Glycopeptides Show that Proteins in High Density Lipoprotein Particles Are Enriched with Specific Glycosylation Compared to Plasma. J. Proteome Res. 17 (2), 834–845. 10.1021/acs.jproteome.7b00604 29212317 PMC6343480

[B14] Kawakami A. Aikawa M. Libby P. Alcaide P. Luscinskas F. W. Sacks F. M. (2006). Apolipoprotein CIII in Apolipoprotein B Lipoproteins Enhances the Adhesion of Human Monocytic Cells to Endothelial Cells. Circulation 113 (5), 691–700. 10.1161/circulationaha.105.591743 16461842

[B15] Kegulian N. C. Ramms B. Horton S. Trenchevska O. Nedelkov D. Graham M. J. (2019). ApoC-III Glycoforms Are Differentially Cleared by Hepatic TRL (Triglyceride-Rich Lipoprotein) Receptors. Atvb 39 (10), 2145–2156. 10.1161/atvbaha.119.312723 PMC6761044 31390883

[B16] Kim G. Weiss S. Weiss J. Levine R. (2015). Methionine Oxidation and Reduction in Proteins. Biochim. Biophys. Acta 1840 (2), 901–905. 10.1016/j.bbagen.2013.04.038 PMC3766491 23648414

[B17] Kim H. J. Moon J. H. Kim H. M. Yun M. R. Jeon B. H. Lee B. (2014). The Hypolipidemic Effect of Cilostazol Can Be Mediated by Regulation of Hepatic Low-Density Lipoprotein Receptor-Related Protein 1 (LRP1) Expression. Metabolism 63 (1), 112–119. 10.1016/j.metabol.2013.09.006 24139096

[B18] Kohan A. B. (2015). Apolipoprotein C-III. Curr. Opin. Endocrinol. Diabetes Obes. 22 (2), 119–125. 10.1097/med.0000000000000136 25692924 PMC4524519

[B19] Koska J. Yassine H. Trenchevska O. Sinari S. Schwenke D. C. Yen F. T. (2016). Disialylated Apolipoprotein C-III Proteoform Is Associated with Improved Lipids in Prediabetes and Type 2 Diabetes. J. Lipid Res. 57 (5), 894–905. 10.1194/jlr.p064816 26945091 PMC4847634

[B20] Lao Y. W. Gungormusler-Yilmaz M. Shuvo S. Verbeke T. Spicer V. Krokhin O. V. (2015). Chromatographic Behavior of Peptides Containing Oxidized Methionine Residues in Proteomic LC-MS Experiments: Complex Tale of a Simple Modification. J. Proteomics 125, 131–139. 10.1016/j.jprot.2015.05.018 26025879

[B21] Larsson M. Allan C. M. Jung R. S. Heizer P. J. Beigneux A. P. Young S. G. (2017). Apolipoprotein C-III Inhibits Triglyceride Hydrolysis by GPIHBP1-Bound LPL. J. Lipid Res. 58 (9), 1893–1902. 10.1194/jlr.m078220 28694296 PMC5580902

[B22] Larsson M. Vorrsjö E. Talmud P. Lookene A. Olivecrona G. (2013). Apolipoproteins C-I and C-III Inhibit Lipoprotein Lipase Activity by Displacement of the Enzyme from Lipid Droplets. J. Biol. Chem. 288 (47), 33997–34008. 10.1074/jbc.m113.495366 24121499 PMC3837139

[B23] Lim J. M. Kim G. Levine R. L. (2019). Methionine in Proteins: It's Not Just for Protein Initiation Anymore. Neurochem. Res. 44 (1), 247–257. 10.1007/s11064-017-2460-0 29327308 PMC6446232

[B24] Mahley R. W. Innerarity T. L. Rall S. C. Weisgraber K. H. (1984). Plasma Lipoproteins: Apolipoprotein Structure and Function. J. Lipid Res. 25 (12), 1277–1294. 10.1016/s0022-2275(20)34443-6 6099394

[B25] Mauger J.-F. Couture P. Bergeron N. Lamarche B. (2006). Apolipoprotein C-III Isoforms: Kinetics and Relative Implication in Lipid Metabolism. J. Lipid Res. 47 (6), 1212–1218. 10.1194/jlr.m500455-jlr200 16495512

[B26] Nedelkov D. (2017). Mass Spectrometric Studies of Apolipoprotein Proteoforms and Their Role in Lipid Metabolism and Type 2 Diabetes. Proteomes 5 (4), 27. 10.3390/proteomes5040027 PMC5748562 29036931

[B27] Nicolardi S. Palmblad M. Dalebout H. Bladergroen M. Tollenaar R. A. E. M. Deelder A. M. (2010). Quality Control Based on Isotopic Distributions for High-Throughput MALDI-TOF and MALDI-FTICR Serum Peptide Profiling. J. Am. Soc. Mass. Spectrom. 21 (9), 1515–1525. 10.1016/j.jasms.2010.05.004 20541438

[B28] Nicolardi S. Van Der Burgt Y. E. M. Dragan I. Hensbergen P. J. Deelder A. M. (2013a). Identification of New Apolipoprotein-CIII Glycoforms with Ultrahigh Resolution MALDI-FTICR Mass Spectrometry of Human Sera. J. Proteome Res. 12 (5), 2260–2268. 10.1021/pr400136p 23527852

[B29] Nicolardi S. van der Burgt Y. E. M. Wuhrer M. Deelder A. M. (2013b). Mapping O -glycosylation of Apolipoprotein C-III in MALDI-FT-ICR Protein Profiles. PROTEOMICS 13 (6), 992–1001. 10.1002/pmic.201200293 23335445

[B30] Oldoni F. van Capelleveen J. C. Dalila N. Wolters J. C. Heeren J. Sinke R. J. (2018). Naturally Occurring Variants in LRP1 (Low-Density Lipoprotein Receptor-Related Protein 1) Affect HDL (High-Density Lipoprotein) Metabolism through ABCA1 (ATP-Binding Cassette A1) and SR-B1 (Scavenger Receptor Class B Type 1) in Humans. Arterioscler. Thromb. Vasc. Biol. 38 (7), 1440–1453. 10.1161/atvbaha.117.310309 29853565 PMC6023722

[B31] Olin-Lewis K. Krauss R. M. La Belle M. Blanche P. J. Barrett P. H. R. Wight T. N. (2002). ApoC-III Content of apoB-Containing Lipoproteins Is Associated with Binding to the Vascular Proteoglycan Biglycan. J. Lipid Res. 43 (11), 1969–1977. 10.1194/jlr.m200322-jlr200 12401896

[B32] Olivieri O. Chiariello C. Martinelli N. Castagna A. Speziali G. Girelli D. (2018). Sialylated Isoforms of Apolipoprotein C-III and Plasma Lipids in Subjects with Coronary Artery Disease. Clin. Chem. Lab. Med. 56 (9), 1542–1550. 10.1515/cclm-2017-1099 29652662

[B33] Palmigiano A. Bua R. O. Barone R. Rymen D. Régal L. Deconinck N. (2017). MALDI-MS Profiling of Serum O -glycosylation and N -glycosylation in COG5-CDG. J. Mass. Spectrom. 52 (6), 372–377. 10.1002/jms.3936 28444691

[B34] Paththinige C. S. Rajapakse J. R. D. K. Constantine G. R. Sem K. P. Singaraja R. R. Jayasekara R. W. (2018). Spectrum of Low-Density Lipoprotein Receptor (LDLR) Mutations in a Cohort of Sri Lankan Patients with Familial Hypercholesterolemia - A Preliminary Report. Lipids Health Dis. 17 (1), 1–7. 10.1186/s12944-018-0763-z 29720182 PMC5932885

[B35] Pollin T. I. Damcott C. M. Shen H. Ott S. H. Shelton J. Horenstein R. B. (2008). A Null Mutation in Human APOC3 Confers a Favorable Plasma Lipid Profile and Apparent Cardioprotection. Science 322 (5908), 1702–1705. 10.1126/science.1161524 19074352 PMC2673993

[B36] Ramms B. Gordts P. L. S. M. (2018). Apolipoprotein C-III in Triglyceride-Rich Lipoprotein Metabolism. Curr. Opin. Lipidol. 29 (3), 171–179. 10.1097/mol.0000000000000502 29547399

[B37] Reyes-Soffer G. Sztalryd C. Horenstein R. B. Holleran S. Matveyenko A. Thomas T. (2019). Effects of APOC3 Heterozygous Deficiency on Plasma Lipid and Lipoprotein Metabolism. Atvb 39 (1), 63–72. 10.1161/atvbaha.118.311476 PMC6309928 30580564

[B38] Rocha N. A. East C. Zhang J. McCullough P. A. (2017). ApoCIII as a Cardiovascular Risk Factor and Modulation by the Novel Lipid-Lowering Agent Volanesorsen. Curr. Atheroscler. Rep. 19 (12), 62. 10.1007/s11883-017-0697-3 29124482

[B39] Rosenson R. S. Brewer H. B. Ansell B. J. Barter P. Chapman M. J. Heinecke J. W. (2016). Dysfunctional HDL and Atherosclerotic Cardiovascular Disease. Nat. Rev. Cardiol. 13 (1), 48–60. 10.1038/nrcardio.2015.124 26323267 PMC6245940

[B40] Shachter N. S. (2001). Apolipoproteins C-I and C-III as Important Modulators of Lipoprotein Metabolism. Curr. Opin. Lipidol. 12 (3), 297–304. 10.1097/00041433-200106000-00009 11353333

[B41] Stadtman E. R. Moskovitz J. Levine R. L. (2003). Oxidation of Methionine Residues of Proteins: Biological Consequences. Antioxid. Redox Signaling 5 (5), 577–582. 10.1089/152308603770310239 14580313

[B42] Taskinen M. R. Packard C. J. Borén J. (2019). Emerging Evidence that ApoC-III Inhibitors Provide Novel Options to Reduce the Residual CVD. Curr. Atheroscler. Rep. 21 (8), 27. 10.1007/s11883-019-0791-9 31111320 PMC6527792

[B43] Trenchevska O. Nelson R. W. Nedelkov D. (2016). Mass Spectrometric Immunoassays in Characterization of Clinically Significant Proteoforms. Proteomes 4 (1), 1623–1633. 10.3390/proteomes4010013 PMC5217360 28248223

[B44] Van Der Burgt Y. E. M. Kilgour D. P. A. Tsybin Y. O. Srzentić K. Fornelli L. Beck A. (2019). Structural Analysis of Monoclonal Antibodies by Ultrahigh Resolution MALDI In-Source Decay FT-ICR Mass Spectrometry. Anal. Chem. 91 (3), 2079–2085. 10.1021/acs.analchem.8b04515 30571088 PMC6365908

[B45] Van Herpt T. T. W. Lemmers R. F. H. Van Hoek M. Langendonk J. G. Erdtsieck R. J. Bravenboer B. (2017). Introduction of the DiaGene Study: Clinical Characteristics, Pathophysiology and Determinants of Vascular Complications of Type 2 Diabetes. Diabetology Metab. Syndr. 9 (1), 1–10. 10.1186/s13098-017-0245-x PMC5477157 28649285

[B46] Wada Y. Okamoto N. (2020). Apolipoprotein C-III O-Glycoform Profiling of 500 Serum Samples by Matrix-Assisted Laser Desorption/ionization Mass Spectrometry for Diagnosis of Congenital Disorders of Glycosylation. J. Mass Spectrom. 56 (4), e4597. 10.1002/jms.4597 32677746

[B47] Wyler Von Ballmoos M. C. Haring B. Sacks F. M. (2015). The Risk of Cardiovascular Events with Increased Apolipoprotein CIII: A Systematic Review and Meta-Analysis. J. Clin. Lipidol. 9 (4), 498–510. 10.1016/j.jacl.2015.05.002 26228667

[B48] Xu Y.-X. Peloso G. M. Nagai T. H. Mizoguchi T. Deik A. Bullock K. (2020). EDEM3 Modulates Plasma Triglyceride Level through its Regulation of LRP1 Expression. IScience 23 (4), 100973. 10.1016/j.isci.2020.100973 32213464 PMC7093811

[B49] Yassine H. N. Trenchevska O. Ramrakhiani A. Parekh A. Koska J. Walker R. W. (2015). The Association of Human Apolipoprotein C-III Sialylation Proteoforms with Plasma Triglycerides. PLoS ONE 10 (12), 1–14. 10.1371/journal.pone.0144138 PMC4669142 26633899

