# Supplementary material for: Large-Scale Analysis of Apolipoprotein CIII Glycosylation by Ultrahigh Resolution Mass Spectrometry
Source: Front Chem. 2021 May 7;9:678883. doi: 10.3389/fchem.2021.678883 (PMC8138127; doi:10.3389/fchem.2021.678883)
Supplement: Supplementary file 1 [file DataSheet1.docx]

**Supporting Information**

**High-throughput MALDI FT-ICR MS for the analysis of apolipoprotein-CIII glycosylation from human plasma**

Daniel Demus^1,2^, Annemieke Naber^3^, Viktoria Dotz^1†^, Bas C. Jansen^1,2^, Marco R. Bladergroen^1^, Jan Nouta^1^, Eric J. G. Sijbrands^3^, Mandy van Hoek^3^, Simone Nicolardi^1^, Manfred Wuhrer^1*^

**^1^** **Leiden University Medical Center, Center for Proteomics and Metabolomics, Leiden, Netherlands**

**^2^ Ludger Ltd., Culham Science Centre, Abingdon, Oxfordshire, United Kingdom**

**^3^ Department of Internal Medicine, Erasmus University Medical Center, Rotterdam, Netherlands**

**Table of Contents**

[**Additional experimental details: MS data processing and statistical analysis** 4](#_Toc59381269)

[**Figure S1.** Degree of apo-CIII oxidation in four different plasma samples (without controlled oxidation) 5](#_Toc59381270)

[**Table S1.** Apolipoprotein-CIII proteoforms detected using the MALDI-FT-ICR MS method 6](#_Toc59381271)

[**Table S2.** Relative intensity (and standard deviation) of the oxidoforms of the most abundant apo-CIII proteoforms in 136 standard plasma samples after controlled oxidation 7](#_Toc59381272)

[**Table S3.** Relative intensity (and standard deviation) of the oxidoforms of the most abundant apo-CIII proteoforms in 771 control plasma samples after controlled oxidation 7](#_Toc59381273)

[**Figure S2.** A and B) Enlargements of MALDI FT-ICR MS spectra in the *m/z*-region of apo-CIII_0a_ oxidoforms 8](#_Toc59381274)

[**Figure S3.** Distributions (boxplots) of the quality control parameters S/N (top left), MME (top right) and IPQ (bottom) in 136 standard plasma samples 9](#_Toc59381275)

[**Figure S4.** Distributions (boxplots) of the quality control parameters S/N (top left), MME (top right) and IPQ (bottom) in 771 control plasma samples 10](#_Toc59381276)

[**Table S4.** Average relative peak intensities and standard diviation (SD) values obtained from the analysis of three plasma samples in duplicate using HCCA and SPA as a MALDI matrix. 11](#_Toc59381277)

[**Table S5.** Quality parameters of four major apo-CIII proteoforms: signal to noise (S/N), isotopic pattern quality (IPQ) and mass measurement error (MME; in part-per-million (ppm)) 12](#_Toc59381278)

[**Table S6.** Associations of apo-CIII sialylation with clinical characteristics. 13](#_Toc59381279)

[**Table S7a.** Subgroup analysis in non-diabetic controls, not using statins or fibrates 14](#_Toc59381280)

[**Table S7b.** Subgroup analysis in non-diabetic controls, not using statins or fibrates 14](#_Toc59381281)

[**Table S8a.** Associations of apo-CIII glycoforms with triglycerides, with and without adjustment for BMI 15](#_Toc59381282)

[**Table S8b.** Associations of apo-CIII glycoforms with triglycerides, with and without adjustment for BMI 15](#_Toc59381283)

[**References** 16](#_Toc59381284)

# **Additional experimental details: MS data processing and statistical analysis**

MS data was converted as *.xy files using DataAnalysis (Bruker, ver. 5.0 SR1) before subsequent processing by MassyTools.^1^ Originally developed for automated pro-cessing of MALDI MS data from released N-glycans, MassyTools was here adjusted to large molecules. Specifically, the modification entailed that the full isotopic envelope is calculated for each molecule, from which the largest contributing isotopes are selected for quantitation until a user-specified threshold (v1.0.2-alpha b180626a). The processing parameters listed below are explained in detail in work by Jansen et al.^1^ All spectra were internally calibrated using the most abundant isotopic peak of oxidoforms of the four major apo-CIII proteoforms (apo-CIII0a+2Ox, apo-CIII0c+2Ox, apo-CIII1+2Ox and apo-CIII2+2Ox; m/z values of 8797.228, 9162.361, 9453.456 and 9744.552, respectively). Data curation was performed in a semi-automated manner using Microsoft Excel and a custom-made script in RStudio (version 1.1.463). Spectra were excluded from further analysis if either calibration by MassyTools failed or if their “Fraction of Analyte Area - Background Area above S/N cut-off” parameter value was below 3*IQR of the mean value). Excluded spectra were spot-checked by visual in-spection to confirm their low quality, especially in case of spectra which were close to the cutoff. Out of each sample spot duplicate, the spectrum with the higher absolute area sum of the four proteoforms was kept for further processing. All analytes of interest had to pass set cut-off values for minimum two quality parameters: signal-to-noise ratio (S/N) of the most-abundant isotopic peak, mass accuracy (MME) and isotopic pattern quality (IPQ). The cut-off val-ues were as follows: S/N ≥ 9, MME ≤ 10 and IPQ ≤ 0.2. Spectra, in which an analyte met two out of three quality parameters, were subjected to additional manual inspection for the presence of interfering species. Peak intensities were measured as peak areas of the most abundant isotopic peak. MassyTools extraction parameters were chosen experimentally for optimal data extraction and were as follows: peak extraction width = 0.3, minimum fraction of total isotopic distribution used for extraction = 0.5, background detection window = 20.

Intra- and inter-plate repeatability were calculated based on 136 plasma standards (VisuCon) distributed over 31 sample plates. For intra-plate repeatability, the mean values of relative peak intensities of apo-CIII0a+2Ox, apo-CIII0c+2Ox, apo-CIII1+2Ox and apo-CIII2+2Ox, standard deviations (SD) and coefficients of variations (CV) were calculated for the standards per sample plate. To assess average intra-plate repeatability, the per-plate CVs were averaged. The per-plate means were averaged, SDs and CVs were calculated over all 31 plates to estimate the inter-plate repeatability. Statistical analyses were performed in SPSS version 25 and data visualization was performed in RStudio (version 1.1.463) and Microsoft Excel. To compare apo-CIII glycosylation in males and females, an independent-samples T-test was performed. The distribution of clinical variables was considered normal when Skewness and Kurtosis were within the range of -1 to +1. The association of apo-CIII glycoforms with age, BMI, HDL-cholesterol, non-HDL-cholesterol, low-density lipoprotein (LDL)-cholesterol, tri-glycerides and total cholesterol was analyzed using univariate linear regression models, where apo-CIII glycoforms were entered as dependent variables. Triglycerides were logarithmically transformed prior to analysis, because of a non-normal distribution, to derive p-values and R-square. To investigate the influence of BMI on the association of apo-CIII glycosylation with triglyceride levels, a multiple linear regression model was used which included BMI and triglycerides as independent variables. P < 0.05 was consid-ered statistically significant.

**
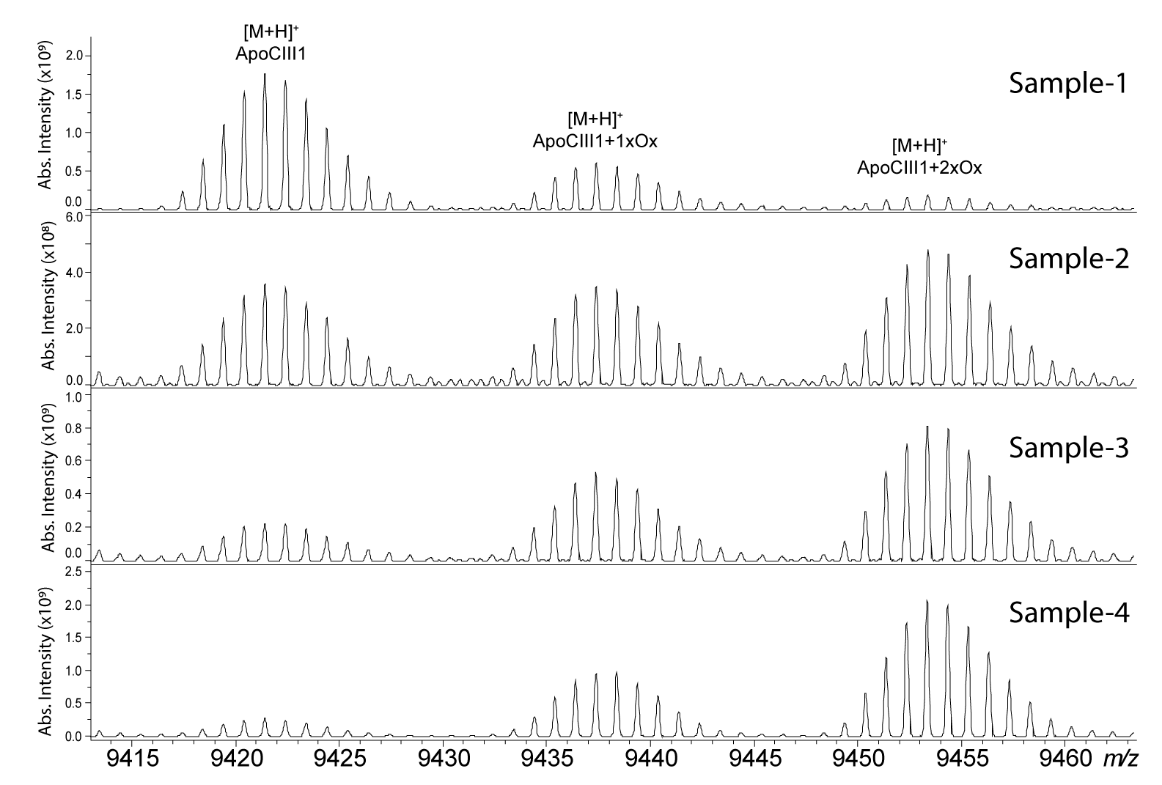
**

**Figure S1.** Degree of apo-CIII oxidation in four different plasma samples (without controlled oxidation). The top spectrum was obtained from a fresh aliquot of plasma sample stored at -80 °C after collection. The other spectra were obtained from plasma aliquots that underwent three freeze/thaw cycles.

**Table S1.** Apolipoprotein-CIII proteoforms detected using the MALDI-FT-ICR MS method. The method allowed to detect four major glycoforms that occur in three different oxido-forms. The combination of glycosylation and oxidation variation results in a total of 12 proteoforms. Detectable C-terminal alanine-cleaved proteoforms are included in the table. TruncA, truncation of a single C-terminal alanine; TruncAA, truncation of two C-terminal alanine residues.

| **Apolipoprotein-CIII amino acid sequence:**  **SEAEDASLLSFMQGYMKHATKTAKDALSSVQESQVAQ- QARGWVTDGFSSLKDYWSTVKDKFSEFWDLDPEVRPTSAVAA** | | |
| --- | --- | --- |
| **Observed apolipoprotein-CIII glycoform** | **Calculated *m/z* value for the most abundant peak within an isotopic distribution**  **[M+H]^+^** | **Ref.** |
| **Apo-CIII_0a_** | 8765.238 | ^2^ |
| **Apo-CIII_0a_+Ox** | 8781.233 | **-** |
| **Apo-CIII_0a_+2Ox** | 8797.228 | **-** |
| **truncA Apo-CIII_0a_+2Ox** | 8726.191 | ^2^ |
| **truncAA Apo-CIII_0a_+2Ox** | 8655.154 | - |
| **Apo-CIII_0C_** | 9130.371 | ^2^ |
| **Apo-CIII_0C_+Ox** | 9146.366 | **-** |
| **Apo-CIII_0C_+2Ox** | 9162.361 | **-** |
| **truncA Apo-CIII_0C_+2Ox** | 9091.323 | ^2^ |
| **truncAA Apo-CIII_0C_+2Ox** | 9019.284 | - |
| **Apo-CIII_1_** | 9421.466 | ^2^ |
| **Apo-CIII_1_+Ox** | 9437.461 | **-** |
| **Apo-CIII_1_+2Ox** | 9453.456 | **-** |
| **truncA Apo-CIII_1_+2Ox** | 9382.419 | ^2,3^ |
| **truncAA Apo-CIII_1_+2Ox** | 9311.382 | - |
| **Apo-CIII_2_** | 9712.562 | ^2,4^ |
| **Apo-CIII_2_+Ox** | 9728.557 | **-** |
| **Apo-CIII_2_+2Ox** | 9744.552 | **-** |
| **truncA Apo-CIII_2_+2Ox** | 9673.514 | ^3^ |
| **truncAA Apo-CIII_2_+2Ox** | 9602.477 | - |
|  |  |  |

**Table S2.** Relative intensity (and standard deviation) of the oxidoforms of the most abundant apo-CIII proteoforms in 136 standard plasma samples after controlled oxidation. The high value of the di-oxidized forms indicates a high oxidation efficiency. The relative intensities may be affected by the presence of interfering peaks as shown in Figure S2.

|  | Non-Ox | Mono-Ox | Di-Ox |
| --- | --- | --- | --- |
| apo-CIII_0a_ | 4.3% (2.0%) | 17.0% (5.0%) | 78.7% (6.4%) |
| apo-CIII_0c_ | 3.3% (0.8%) | 11.6% (1.0%) | 85.1% (1.7%) |
| apo-CIII_1_ | 1.2% (0.4%) | 6.6% (0.8%) | 92.2% (1.0%) |
| apo-CIII_2_ | 0.7% (0.4%) | 6.5% (0.9%) | 92.8% (1.0%) |

**Table S3.** Relative intensity (and standard deviation) of the oxidoforms of the most abundant apo-CIII proteoforms in 771 control plasma samples after controlled oxidation. The high value of the di-oxidized forms indicates a high oxidation efficiency. The relative intensities of non- and mono-oxidized apo-CIII_0a_ and apo-CIII_0c_ were increased as a result of the presence of interfering species as exemplified in Figure S2.

|  | Non-Ox | Mono-Ox | Di-Ox |
| --- | --- | --- | --- |
| apo-CIII_0a_ | 8.8% (6%) | 22.7% (12.3%) | 68.4% (16.3%) |
| apo-CIII_0c_ | 4.3% (4.3%) | 11.6% (1.8%) | 84.1% (5.0%) |
| apo-CIII_1_ | 2.0% (0.6%) | 7.4% (0.9%) | 90.6% (1.2%) |
| apo-CIII_2_ | 1.7% (1.1%) | 6.3% (1.4%) | 91.9% (1.7%) |


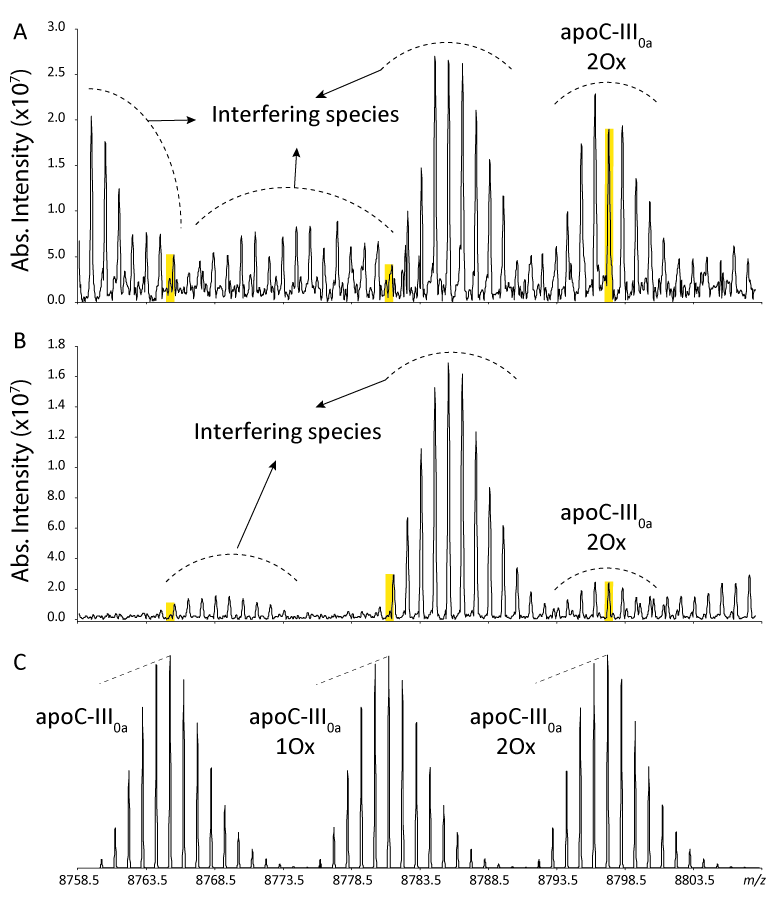


**Figure S2.** A and B) Enlargements of MALDI FT-ICR MS spectra in the *m/z*-region of apo-CIII_0a_ oxidoforms. The presence of interfering species can affect the determination of the intensity of the most intense isotopic peaks which is calculated in the regions of the spectra highlighted in yellow. In fact, the high relative abundance of Non-Ox and Mono-Ox apo-CIII0a forms (namely 8.8% and 22.7%) in Table S3 was attributed to the presence of interfering species rather than an incomplete oxidation. C) Theoretical isotopic distributions.


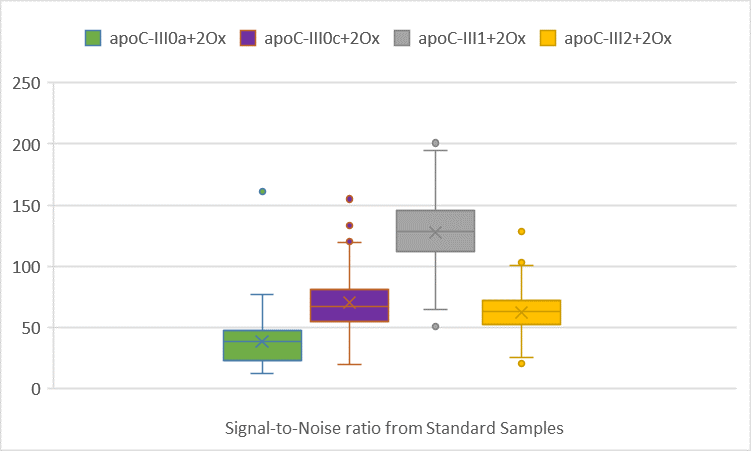

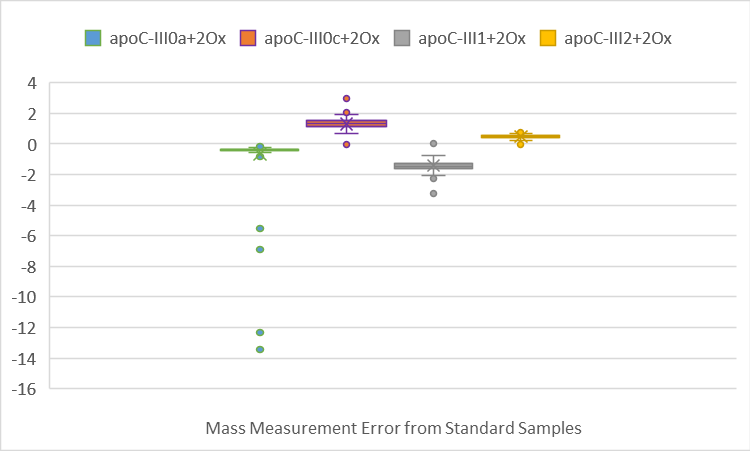

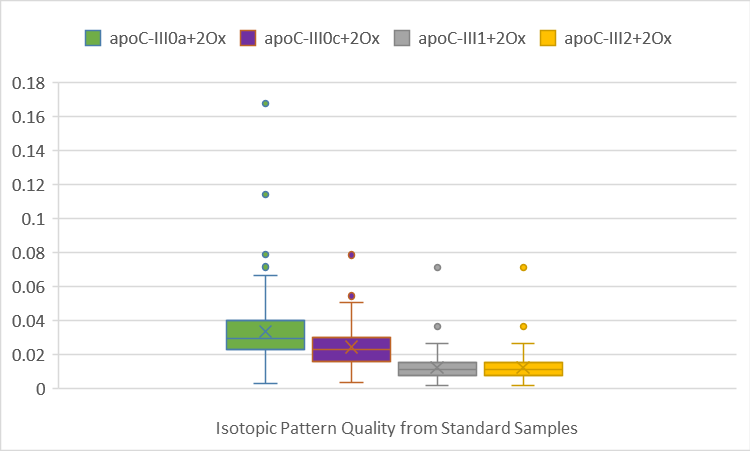


|  |  |  |  |
| --- | --- | --- | --- |

**Figure S3.** Distributions (boxplots) of the quality control parameters S/N (top left), MME (top right) and IPQ (bottom) in 136 standard plasma samples.

**
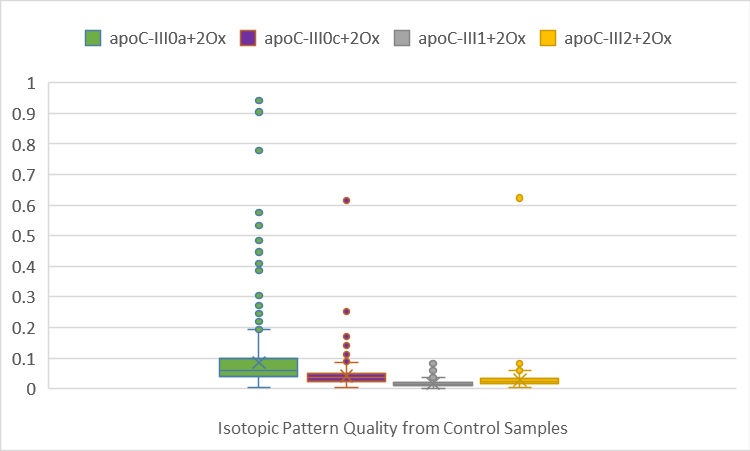

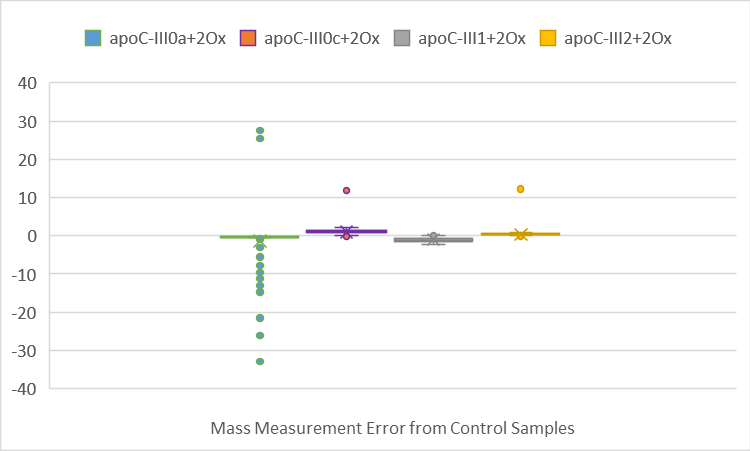

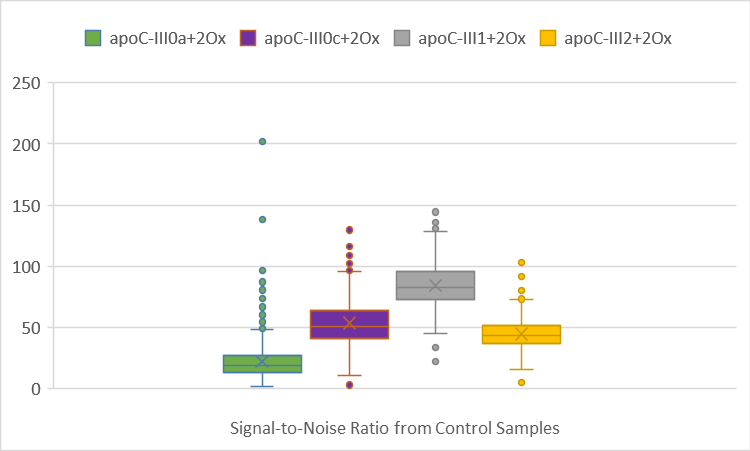
**

**Figure S4.** Distributions (boxplots) of the quality control parameters S/N (top left), MME (top right) and IPQ (bottom) in 771 control plasma samples.

**Table S4.** Average relative peak intensities and standard diviation (SD) values obtained from the analysis of three plasma samples in duplicate using HCCA and SPA as a MALDI matrix.

|  |  | **apo-CIII_0a_** | **apo-CIII_0c_** | **apo-CIII_1_** | **apo-CIII_2_** |
| --- | --- | --- | --- | --- | --- |
| **HCCA** | Relative Intensity | 4% | 19% | 67% | 10% |
|  | SD | 0% | 2% | 1% | 1% |
|  |  |  |  |  |  |
| **SPA** | Relative Intensity | 4% | 11% | 64% | 21% |
|  | SD | 0% | 1% | 2% | 1% |

**Table S5.** Quality parameters of four major apo-CIII proteoforms: signal to noise (S/N), isotopic pattern quality (IPQ) and mass measurement error (MME; in part-per-million (ppm)). The average values and standard deviations (SD) were calculated based on 136 VisuCon plasma standards and 771 controls used in the cohort study. H_2_O_2_ treatment provided good quality spectra with analyte peaks meeting minimum two of the set quality criteria: PPM ≤ 10, IPQ ≤ 0.2 and S/N ≥ 9.

|  |  | | **Apo-CIII_0a_+2Ox**  ***m/z* = 8797.228** | **Apo-CIII_0c_+2Ox**  ***m/z* = 9162.361** | **Apo-CIII_1_+2Ox**  ***m/z* = 9453.456** | **Apo-CIII_2_+2Ox**  ***m/z* = 9744.552** |
| --- | --- | --- | --- | --- | --- | --- |
| **Plasma standards**  (n = 136) | **S/N** | **Mean** | **38.37** | **70.29** | **127.48** | **62.42** |
|  |  | SD | 17.80 | 21.45 | 27.69 | 17.11 |
|  | **IPQ** | **Mean** | **0.03** | **0.02** | **0.01** | **0.02** |
|  |  | SD | 0.02 | 0.01 | 0.01 | 0.01 |
|  | **MME (ppm)** | **Mean** | **0.65** | **1.31** | **1.41** | **0.48** |
|  |  | SD | 1.66 | 0.37 | 0.39 | 0.13 |
|  |  |  |  |  |  |  |
| **Controls**  (n = 771) | **S/N** | **Mean** | **22.03** | **53.59** | **84.45** | **44.86** |
|  |  | SD | 15.18 | 17.96 | 17.13 | 10.84 |
|  | **IPQ** | **Mean** | **0.09** | **0.04** | **0.02** | **0.03** |
|  |  | SD | 0.09 | 0.03 | 0.01 | 0.03 |
|  | **MME (ppm)** | **Mean** | **1.64** | **0.98** | **1.04** | **0.37** |
|  |  | SD | 4.36 | 0.6 | 0.5 | 0.46 |

**Table S6.** Associations of apo-CIII sialylation with clinical characteristics. The sum of the glycoforms Apo-CIII_0c_, Apo-CIII_1_, and Apo-CIII_2_ was set to 1.0.

| Characteristics | Apo-CIII_0c_ | | | | Apo-CIII_1_ | | | | Apo-CIII_2_ | | | |
| --- | --- | --- | --- | --- | --- | --- | --- | --- | --- | --- | --- | --- |
|  | Male | Female | | p-value | Male | Female | | p-value | Male | Female | | p-value |
| Sex (mean ± SD) | 0.114  ± 0.026 | 0.116  ± 0.025 | | 0.531 | 0.667  ± 0.028 | 0.674  ± 0.031 | | **0.001** | 0.218  ± 0.036 | 0.209  ± 0.039 | | **0.003** |
|  | beta | | p-value | | beta | | p-value | | beta | | p-value | |
| Age | -9.22E-05 | | 5.05E-01 | | -2.96E-04 | | 7.06E-02 | | 4.06E-04 | | 5.11E-02 | |
| BMI | 8.34E-04 | | **8.15E-04** | | 1.55E-03 | | **1.16E-07** | | -2.42E-03 | | **6.13E-11** | |
| HDL cholesterol | -1.09E-03 | | 6.80E-01 | | -6.35E-03 | | **4.21E-02** | | 7.66E-03 | | 5.30E-02 | |
| non-HDL cholesterol | 1.39E-03 | | 1.64E-01 | | 7.60E-03 | | **6.54E-11** | | -9.00E-03 | | **1.11E-09** | |
| LDL cholesterol | 1.66E-03 | | 1.20E-01 | | 7.55E-03 | | **1.59E-09** | | -9.23E-03 | | **6.45E-09** | |
| Triglycerides | 1.92E-03 | | 1.01E-01 | | 1.50E-02 | | **1.99E-20** | | -1.69E-02 | | **8.31E-17** | |
| Total cholesterol | 1.18E-03 | | 2.26E-01 | | 6.42E-03 | | **2.01E-08** | | -7.59E-03 | | **1.75E-07** | |

Blue: negative associations, red: positive associations, bold: significant p-value. P-values of logarithmically transformed triglyceride concentrations, beta of non-transformed concen-trations.

**Table S7a.** Subgroup analysis in non-diabetic controls, not using statins or fibrates. The sum of the glycoforms Apo-CIII_0a_, Apo-CIII_0c_, Apo-CIII_1_, and Apo-CIII_2_ was set to 1.0.

|  | Apo-CIII_0a_ | | Apo-CIII_0c_ | | Apo-CIII_1_ | | Apo-CIII_2_ | |
| --- | --- | --- | --- | --- | --- | --- | --- | --- |
|  | beta | p-value | beta | p-value | beta | p-value | beta | p-value |
| Age | 2.37E-05 | 8.44E-01 | -1.18E-04 | 3.84E-01 | -2.63E-04 | 1.02E-01 | 3.60E-04 | 1.04E-01 |
| BMI | -5.76E-04 | **8.24E-03** | 6.48E-04 | **8.19E-03** | 1.82E-03 | **1.86E-10** | -1.91E-03 | **1.49E-06** |
| HDL cholesterol | 1.46E-03 | 5.26E-01 | 5.45E-05 | 9.83E-01 | -9.24E-03 | **2.20E-03** | 7.67E-03 | 6.56E-02 |
| non-HDL cholesterol | 9.09E-04 | 3.19E-01 | 1.86E-03 | 6.75E-02 | 6.23E-03 | **1.55E-07** | -8.85E-03 | **6.12E-08** |
| LDL cholesterol | 1.08E-03 | 2.72E-01 | 2.51E-03 | **2.25E-02** | 6.14E-03 | **1.80E-06** | -9.56E-03 | **6.63E-08** |
| Triglycerides | 2.12E-03 | **4.65E-02** | 7.37E-04 | 4.89E-01 | 1.19E-02 | **7.20E-14** | -1.46E-02 | **4.68E-12** |
| Total cholesterol | 1.13E-03 | 2.14E-01 | 1.86E-03 | 6.72E-02 | 4.74E-03 | **6.65E-05** | -7.59E-03 | **3.40E-06** |

Blue: negative associations, red: positive associations, bold: significant p-value. P-values of logarithmically transformed triglyceride concentrations, beta of non-transformed concentrations.

**Table S7b.** Subgroup analysis in non-diabetic controls, not using statins or fibrates. The sum of the glycoforms Apo-CIII_0c_, Apo-CIII_1_, and Apo-CIII_2_ was set to 1.0.

|  | Apo-CIII_0c_ | | Apo-CIII_1_ | | Apo-CIII_2_ | |
| --- | --- | --- | --- | --- | --- | --- |
|  | beta | p-value | beta | p-value | Beta | p-value |
| Age | -1.20E-04 | 4.07E-01 | -2.49E-04 | 1.51E-01 | 3.81E-04 | 8.52E-02 |
| BMI | 5.97E-04 | **2.25E-02** | 1.47E-03 | **1.96E-06** | -2.13E-03 | **7.33E-08** |
| HDL cholesterol | 1.96E-04 | 9.43E-01 | -8.52E-03 | **8.87E-03** | 8.50E-03 | **4.10E-02** |
| non-HDL cholesterol | 1.99E-03 | 6.74E-02 | 7.04E-03 | **3.80E-08** | -9.05E-03 | **2.96E-08** |
| LDL cholesterol | 2.69E-03 | **2.19E-02** | 6.99E-03 | **4.41E-07** | -9.73E-03 | **3.67E-08** |
| Triglycerides | 9.42E-04 | 4.00E-01 | 1.41E-02 | **4.21E-17** | -1.49E-02 | **2.14E-12** |
| Total cholesterol | 2.01E-03 | 6.43E-02 | 5.66E-03 | **1.01E-05** | -7.66E-03 | **2.73E-06** |

Blue: negative associations, red: positive associations, bold: significant p-value.
P-values of logarithmically transformed triglyceride concentrations, beta of non-transformed concentrations.

**Table S8a.** Associations of apo-CIII glycoforms with triglycerides, with and without adjustment for BMI. The sum of the glycoforms Apo-CIII0a, Apo-CIII0c, Apo-CIII1, and Apo-CIII2 was set to 1.0.

|  | Apo-CIII_0a_ | | | Apo-CIII_0c_ | | | Apo-CIII_1_ | | | Apo-CIII_2_ | | |
| --- | --- | --- | --- | --- | --- | --- | --- | --- | --- | --- | --- | --- |
|  | beta | p-value | R2 | beta | p-value | R2 | beta | p-value | R2 | beta | p-value | R2 |
| Triglycerides | 2.61E-03 | **7.45E-03** | 0.01 | 1.60E-03 | 1.53E-01 | 0.00 | 1.25E-02 | **9.02E-16** | 0.09 | -1.66E-02 | **1.44E-16** | 0.09 |
| Triglycerides adjusted for BMI | 3.69E-03 | **3.64E-04** | 0.02 | 1.66E-05 | 7.96E-01 | 0.02 | 1.05E-02 | **3.70E-11** | 0.11 | -1.43E-02 | **2.72E-12** | 0.09 |

Blue: negative associations, red: positive associations, bold: significant p-value. P-value and R-square (R2) are calculated for the logarithmically transformed values of triglycerides. 'Triglycerides adjusted for BMI' represent a multivariate linear model, BMI and triglycerides as independent variables.

**Table S8b.** Associations of apo-CIII glycoforms with triglycerides, with and without adjustment for BMI. The sum of the glycoforms Apo-CIII0c, Apo-CIII1, and Apo-CIII2 was set to 1.0.

|  | Apo-CIII_0c_ | | | Apo-CIII_1_ | | | Apo-CIII_2_ | | |
| --- | --- | --- | --- | --- | --- | --- | --- | --- | --- |
|  | beta | p-value | R2 | beta | p-value | R2 | beta | p-value | R2 |
| Triglycerides | 1.92E-03 | 1.01E-01 | 0.00 | 1.50E-02 | **1.99E-20** | 0.12 | -1.69E-02 | **8.31E-17** | 0.10 |
| Triglycerides adjusted for BMI | 4.12E-04 | 5.54E-01 | 0.01 | 1.38E-02 | **1.10E-16** | 0.12 | -1.42E-02 | **4.78E-12** | 0.11 |

Blue: negative associations, red: positive associations, bold: significant p-value. P-value and R-square (R2) are calculated for the logarithmically transformed values of triglycerides. 'Triglycerides adjusted for BMI' represent a multivariate linear model, BMI and triglycerides as independent variables.

# **References**

(1) Jansen, B. C.; Reiding, K. R.; Bondt, A.; Hipgrave Ederveen, A. L.; Palmblad, M.; Falck, D.; Wuhrer, M. MassyTools: A High-Throughput Targeted Data Processing Tool for Relative Quantitation and Quality Control Developed for Glycomic and Glycoproteomic MALDI-MS. *J. Proteome Res.* **2015**, *14* (12), 5088–5098. https://doi.org/10.1021/acs.jproteome.5b00658.

(2) Nicolardi, S.; van der Burgt, Y. E. M.; Wuhrer, M.; Deelder, A. M. Mapping O -Glycosylation of Apolipoprotein C-III in MALDI-FT-ICR Protein Profiles. *Proteomics* **2013**, *13* (6), 992–1001. https://doi.org/10.1002/pmic.201200293.

(3) Nelsestuen, G. L.; Zhang, Y.; Martinez, M. B.; Key, N. S.; Jilma, B.; Verneris, M.; Sinaiko, A.; Kasthuri, R. S. Plasma Protein Profiling: Unique and Stable Features of Individuals. *Proteomics* **2005**, *5* (15), 4012–4024. https://doi.org/10.1002/pmic.200401234.

(4) Nicolardi, S.; Van Der Burgt, Y. E. M.; Dragan, I.; Hensbergen, P. J.; Deelder, A. M. Identification of New Apolipoprotein-CIII Glycoforms with Ultrahigh Resolution MALDI-FTICR Mass Spectrometry of Human Sera. *J. Proteome Res.* **2013**, *12* (5), 2260–2268. https://doi.org/10.1021/pr400136p.
